# Inhibition of Cancer Derived Cell Lines Proliferation by Synthesized Hydroxylated Stilbenes and New Ferrocenyl-Stilbene Analogs. Comparison with Resveratrol

**DOI:** 10.3390/molecules19067850

**Published:** 2014-06-11

**Authors:** Malik Chalal, Dominique Delmas, Philippe Meunier, Norbert Latruffe, Dominique Vervandier-Fasseur

**Affiliations:** 1 Université de Bourgogne, 21000 Dijon, France; 2 Institut de Chimie Moléculaire de l’Université de Bourgogne, ICMUB UMR CNRS 6302, 9, avenue Alain Savary, 21000 Dijon, France; 3 Laboratoire de Biochimie (Bio-PeroxIL) INSERM IFR 100, 6, boulevard Gabriel, Dijon, France; 4 INSERM UMR 866, 7, boulevard Jeanne d’Arc, 21000 Dijon, France

**Keywords:** resveratrol, methoxystilbenes, ferrocenylstilbene analogs, colon cancer, hepatoblastoma

## Abstract

Further advances in understanding the mechanism of action of resveratrol and its application require new analogs to identify the structural determinants for the cell proliferation inhibition potency. Therefore, we synthesized new *trans*-resveratrol derivatives by using the Wittig and Heck methods, thus modifying the hydroxylation and methoxylation patterns of the parent molecule. Moreover, we also synthesized new ferrocenylstilbene analogs by using an original protective group in the Wittig procedure. By performing cell proliferation assays we observed that the resveratrol derivatives show inhibition on the human colorectal tumor SW480 cell line. On the other hand, cell viability/cytotoxicity assays showed a weaker effects on the human hepatoblastoma HepG2 cell line. Importantly, the lack of effect on non-tumor cells (IEC18 intestinal epithelium cells) demonstrates the selectivity of these molecules for cancer cells. Here, we show that the numbers and positions of hydroxy and methoxy groups are crucial for the inhibition efficacy. In addition, the presence of at least one phenolic group is essential for the antitumoral activity. Moreover, in the series of ferrocenylstilbene analogs, the presence of a hidden phenolic function allows for a better solubilization in the cellular environment and significantly increases the antitumoral activity.

## 1. Introduction

**Figure 1:**
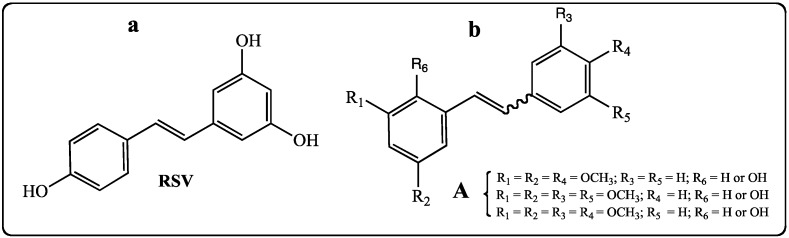
(**a**) Structure of *trans*-resveratrol (**RSV**). (**b**) Structure of *cis* and *trans*-resveratrol derivatives.

Polyphenolic compounds, including stilbenes, anthocyans, catechins and their oligomers, are widespread in a large number of plants. Polyphenolic stilbenoids have been discovered in numerous species, for instance, in the roots of the Asiatic plant *Polygonum cuspidatum* [1], in the South African plant *Erythrophleum lasianthu* [2], in red fruit, including grapes [3,4,5], in red wine [6,7], in Itadori green tea [8], in peanuts [9], and in rhubarb [10]. The common feature of these different plants is the presence of a phytoalexin, *trans*-resveratrol or *trans*-3,5,4'-trihydroxystilbene (**RSV**, Figure 1a) [1,3,4,5,8,9,11,12]. This well-known polyphenol proves to be a true (Swiss Army knife) molecule [13] in the therapeutic and biological fields [14,15,16]. Indeed, numerous publications and reviews report about *trans*-resveratrol’s antitumoral [17,18], anti-inflammatory [19], antiviral [20], antimicrobial [21], and antifungal [22,23,24] activities. In addition, *trans*-resveratrol is a neuroprotective agent [25,26] and can also prevent heart disease [27,28,29]. The antioxidant features of *trans*-resveratrol may partly explain these numerous activities [30,31,32]. In cancer research, it has been shown that involvement of *trans*-resveratrol in antitumoral activity is also due to its ability to bind different cellular targets [33,34]. However, several derivatives of *trans*-resveratrol show a better activity than the parent molecule towards specific types of cancer [35]. The modifications of the chemical structure of *trans*-resveratrol involve the number and the position of the phenolic groups [35,36,37], the presence on the aromatic rings of methoxy groups [38,39,40,41], long alkyl chains [38,42], or functionalized chains [43]. These structural modifications improve mostly the lipophilicity of the stilbenes in the cellular environment and thus their biological effects inside the cell [44]. However, the methoxylated derivatives of *trans*-resveratrol seem to have a different way of delaying cancer growth. Indeed, our group has studied the biological activities of *E-* and *Z*-methoxylated stilbenes against the human colorectal tumor SW480 cell line and has reported that the methoxy group is a determinant substitution for the molecules bearing a *Z* configuration in inhibition of this cell line (compounds **A**, Figure 1) [45].

Zhang *et al.* have confirmed that *trans*-resveratrol was known to be active only in its *E* configuration while some methoxylated derivatives proved to be active in the *Z* configuration [41]. In order to deepen our understanding of the mechanism of action and to highlight compounds with enhanced effects on colorectal tumor SW480 and hepatoblastoma HepG2 cell lines, we synthesized a series of *E*-stilbenes, including three new original ferrocenylstilbene analogs, by improved Wittig and Heck methods [46]. Each compound was submitted to evaluation for biological properties (antiproliferative activity and cell cycle disturbance of SW480 colon cancer and hepatic HepG2 cancer cells). To obtain an inhibitory effect, the chemical parameters studied are the following: (a) the presence of a hydroxy group in position 4; (b) the increased effect due to the presence of a methoxy group (a decrease of the polar character leading to an increase in lipophilic property); (c) the lack (or masked form) of other hydroxy groups. In the series of ferrocenylstilbene analogs, the presence of a phenolic function as an ester greatly increases the antitumoral activity. Most of synthetic compounds are more efficient towards colorectal SW480 cells than liver-derived HepG2 cells. Furthermore, the lack of effects on non-tumor cells (IEC18 intestinal epithelium cells) demonstrates the selectivity of these molecules for cancer cells, which is an important aspect for possible therapeutic applications.

## 2. Results and Discussion

### 2.1. Chemical Results

#### 2.1.1. Synthesis of *E*-4-Hydroxystilbenes

**Figure 2:**
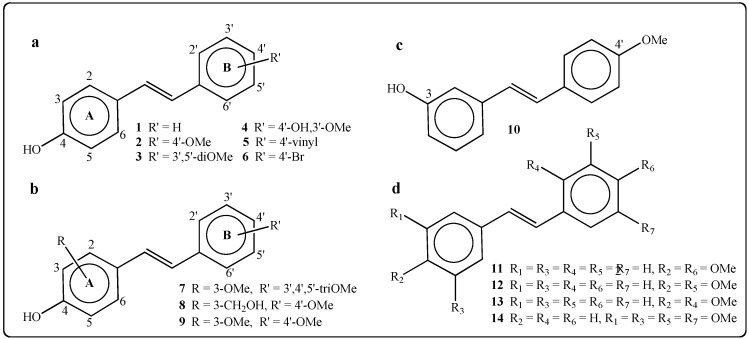
Molecular structure of synthetic stilbene derivatives. (**a**) 4-OH stilbenes bearing substituents on cycle B. (**b**) 4-OH stilbenes bearing substituents on cycle A and/or cycle B. (**c**) 3-hydroxy-4'-methoxystilbene (**10**). (**d**) Stilbenes without free phenolic function.

Given the importance of the free phenolic function in position 4 [30,31], we focused on the preparation of derivatives bearing a free phenolic group in position 4 and substituents on the ring B of the stilbenes (compounds **1**–**6**; Figure 2a) or on the A and B rings of the stilbenes (compounds **7**–**9**; Figure 2b). The methoxy group was often chosen as a substituent to improve the membrane permeability of the stilbenes. To highlight the importance of the presence and the position of the phenolic function in the activity of the stilbenes towards tumor cell lines, one derivative with OH group in position 3 was prepared (compound **10**; Figure 2c) and four resveratrol analogs without a free phenolic function were synthesized (compounds **11**–**14**; Figure 2d). Compound **10** was already studied by Zhang *et al.* for its effects on NQO1 induction in hepatoma cells, but its synthesis was not described [41].

On the contrary, compounds **1**–**4**, **6**, **7**, **12** and **13** were already synthesized by different method, including Horner-Emmons-Haworth [35,47,48], Perkin [49,50,51] and Mizoroki-Heck reactions [52]. Previously, our group has reported the synthesis of compounds **1**–**14** by two standard methods [46]. Stilbenes **4**, **7**–**13** were prepared by palladium-catalyzed Heck coupling using ferrocenylphosphane ligands. In our protocol, the hydroxylated stilbenes were obtained without the need of protection/deprotection steps on the phenolic functions. Stilbenes **1**–**3**, **5**, **6** and **14** were prepared by Wittig reactions; the protection on the hydroxy groups of aromatic aldehydes was achieved using the labile trimethylsilyl group, rarely used in this case. This protective group was easily cleaved during the aqueous work-up following the Wittig reaction. 

#### 2.1.2. Synthesis of Stilbenes Bearing Ferrocenylstilbene Analogs

**Figure 3:**
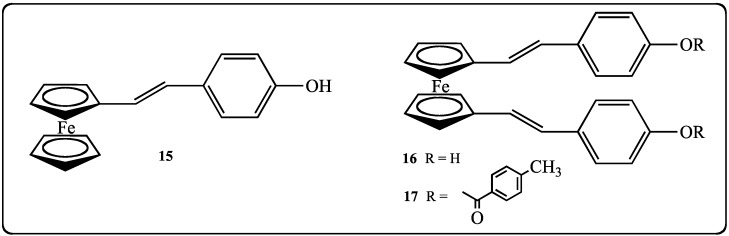
Molecular structure of ferrocenyl-stilbene analogs **15**–**17**.

In addition to these stilbenes bearing classical substituents, we developed original ferrocenyl-analogs of stilbenes **15**–**17** (Figure 3). 

**Figure 4:**
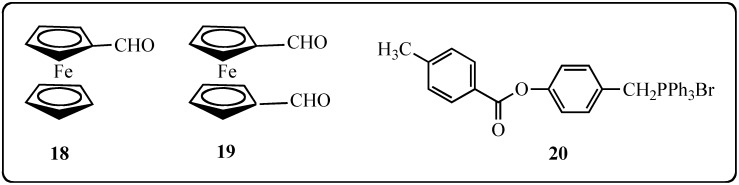
Starting reagents for the preparation of ferrocenyl-stilbene analogs **18**–**20**.

Indeed, since the discovery of the antitumoral properties of cisplatin [53], the therapeutic interests in metallic complexes and organometallic compounds has increased steadily [54], especially for ferrocenyl derivatives [55]. Several organometallic compounds bearing a ferrocenyl group display better biological properties than their organic counterparts, such as chloroquine and ferroquine used in the treatment of malaria [56]. A key example of an anticancer ferrocene derivative is the anti-breast cancer ferrocifen series. Jaouen’s group has synthesized different derivatives of the ferrocen complexes of tamoxifen and has shown complementary activities of these compounds [57,58]. Therefore, in the aim to improve the antitumoral activities of the polyphenols, we have targeted the synthesis of an original stilbene molecular structure wherein a ferrocenyl ring replaced a benzenic ring; the position 4 of the remaining benzenic ring was substituted by a free phenolic function. The proposed strategy to access this series of ferrocenylstilbene analogs is to react under Wittig reaction conditions ferrocenecarbaldehyde (**18**) or ferrocene-1,1'-dicarbaldehyde (**19**) [59] with a benzylphosphonium bromide bearing a protected phenolic function **20** (Figure 4).

**Scheme 1:**
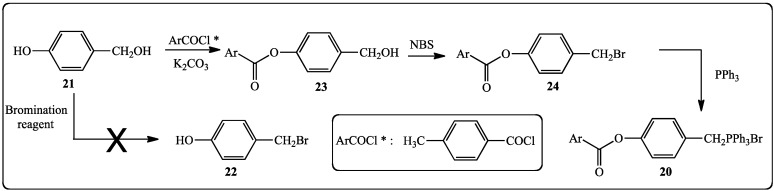
Synthesis of benzylphosphonium bromide **20**.

The precursor of **20** is 4-hydroxybenzylic alcohol (**21**), the corresponding bromide **22** is not commercially available and cannot be prepared by bromination of **21** because of its instability [60] (Scheme 1). Thus, the protection of the phenolic function has to be carried out before the bromination of the benzylic alcohol and in addition, the protective group should be stable to the bromination reagent. These conditions preclude the use of the trimethylsilyl group [46]. Therefore, the phenolic function has been protected as an ester function by reacting **21** with *para*-toluoyl chloride in the presence of K_2_CO_3_ and acetone as a solvent [61]. The benzylphosphonium bromide **20** was obtained by reacting benzylic alcohol **23** successively with N-bromosuccinimide in CH_2_Cl_2_ [62] and triphenylphosphine in toluene (Scheme 1). 

**Scheme 2:**
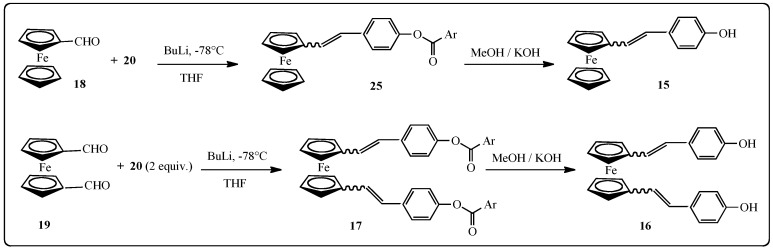
Synthesis of ferrocenyl-stilbene analogs **15**–**17**.

Finally, the benzylphosphonium bromide **20** was reacted with ferrocenecarbadehyde (**18**) in the presence of butyl lithium in THF. The cleavage of phenolic esters was carried out by KOH in methanol [63] and the ferrocenylstilbene analog **15** was recovered in 52% yield. In the same manner, the ferrocenyl derivative was obtained from **20** and ferrocene-1,1'-dicarbaldehyde (**19**) in 47% yield (Scheme 2).

### 2.2. Biological Effects

We compared the potency of the new resveratrol synthetic analogs towards the human colorectal tumor cell line SW480, the human hepatoblastoma HepG2 cell line and the rat normal intestine epithelium IEC18 cell, comparing their effect with the natural reference molecule, *i.e.*, *trans*-resveratrol. 

#### 2.2.1. Effect of Stilbene Derivatives on Human Colorectal Tumor SW 480 Cell Line Proliferation

**Figure 5:**
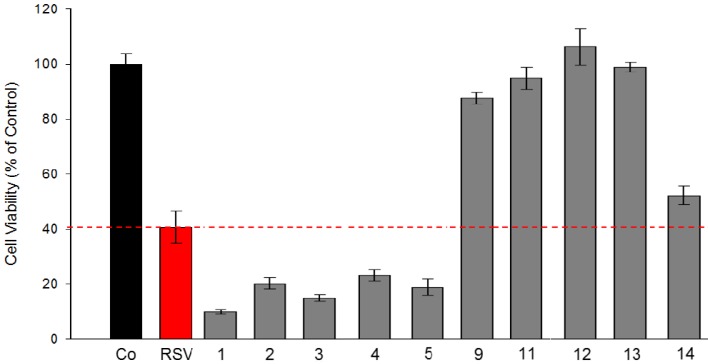
Effect of stilbene derivatives on human cancerous colorectal SW480 cell viability. Cells were grown for 48 h in the presence of 30 µM resveratrol (or no RSV in a control experiment) or 30 µM stilbene derivatives (numbered on the x-axis). Cell viability was determined by counting cells using the trypan blue test (Co: cells control test). Data correspond to the mean of two independent experiments.

Firstly, we have determined the sensitivity of human tumoral colorectal cell line SW480 towards the newly synthesized stilbene derivatives and compared them to resveratrol, the parent molecule. Figure 5 shows, as expected and in agreement with the literature [64], that resveratrol at 30 µM decreases drastically cell viability which is of 40% compared to the control (Figure 5).

Interestingly, compounds **1**–**5** exhibit higher cytotoxicity than resveratrol. These derivatives bear, like resveratrol, at least one phenol group in the para position of the stilbene ring. The only structural differences between these molecules are the positions and numbers of methoxy groups. The efficiency of compound **1** indicates that its activity is due to the phenolic group, despite the absence of methoxy groups on its skeleton. Compound **14**, a tetramethoxylated derivative, shows similar activity as resveratrol, suggesting that these substituents are not essential for the activity. However, the fact that compounds **9**, **11**–**13** have only weak effects seems to indicate that a free phenolic group in the *para* position of the aromatic ring is needed for toxicity. 

#### 2.2.2. Effect of Stilbene Derivatives on the Cell Cycle Phase of the SW480 Cell Line

**Figure 6:**
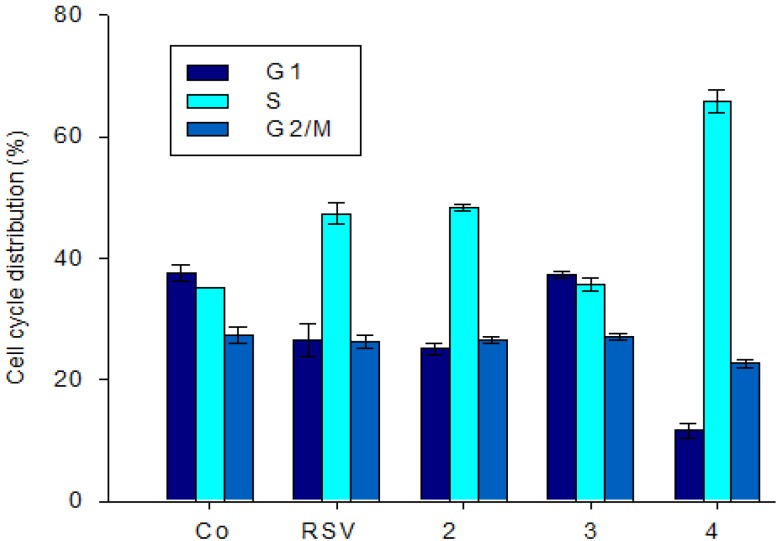
Influence of stilbene derivatives on the cell cycle phases of the SW480 cells line. Cells were grown for 48 h in the presence of 30 µM resveratrol (or no RSV in a control experiment) or 30 µM stilbene derivatives (numbered on the x-axis). After treatment, nuclear DNA was labeled with propidium iodide. The cell cycle effect of the tested compounds was done analysing cell distribution in the different phases of the cell cycle (mean ± standard deviation of two independent experiments).

To further explore the mechanisms by which the most efficient compounds exert their antiproliferative potencies, we studied their effects on the cell cycle distribution of SW480 cells (Figure 6). The treatment of cells with compound **2**, which bears a hydroxy group in position 4 and a methoxy group in position 4', induces an accumulation of SW480 cells in S phase in the same manner as resveratrol (Figure 6). Interestingly, compound **4**, bearing hydroxy groups at positions 4 and 4' and a methoxy group at position 3, leads to an increase of S phase which is better than that of resveratrol and compound **2**. In contrast, pterostilbene (**3**) does not show any effect on the cell cycle, while it inhibits cell proliferation. This derivative has been reported to induce a blockade of HL60 intestine cancer cells in the G_1_ phase, and to induce apoptosis [65]. The distribution of cells in the different cell cycle phases is reported in Figure 1 of the supplementary material.

One of the mechanisms by which resveratrol modulates carcinogenesis is the blockage of cells in S phase [66]. However, these effects at the cell cycle are complex and depend on the cell type, the resveratrol concentration and the duration of the treatment. Indeed, a low concentration of resveratrol induces accumulation of cells in S phase while at higher concentrations it leads to cell accumulation in G_1_ or G_2_/M phases [67]. Moreover, many cytotoxic agents also induce cell death by apoptosis. We have previously shown in SW480 and in HepG2 cell lines that resveratrol induces accumulation of cells in early S phase by action on the p21 protein and on the cyclin/cdk complexes formation and activity [68]. In the structural core of resveratrol, the phenol group in position 4 would be responsible for the antiproliferative effect by its action on DNA polymerases alpha and gamma [69,70]. Indeed, the increase of number of hydroxy groups on the stilbene moiety of resveratrol derivatives led to an increase of inhibition of tumor cell proliferation [71]. On the other hand, She *et al.* [72] have shown that *trans*-3,3',4',5-tetrahydroxystilbene and *trans*-3,3',4',5,5'-pentahydroxystilbene exhibit a higher apoptotic effect than resveratrol on the epidermal JB6 cell line.

#### 2.2.3. Evaluation of Toxicity Level of Stilbene Derivatives Towards Non-Cancerous Intestinal Epithelial Cells

**Figure 7:**
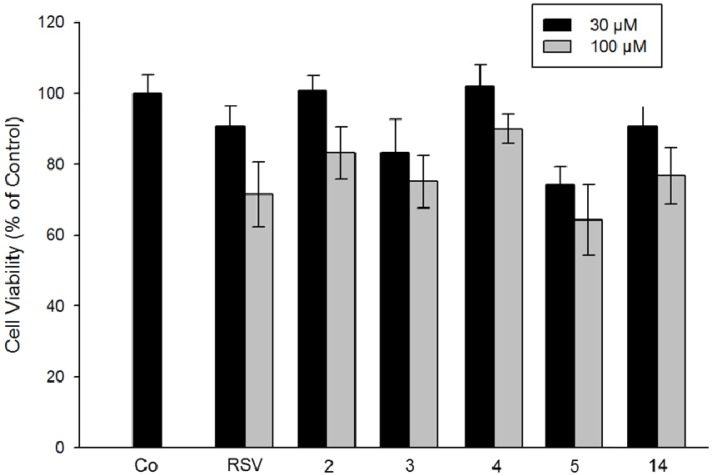
Effect of stilbenes derivatives on the proliferation of non-transformed IEC18 cells. Cells were grown for 48 h in the presence of 30 µM resveratrol (or no RSV in a control experiment) or 30 µM and 100 µM stilbene derivatives (numbered on the x-axis). Cell viability was determined by counting cells using the trypan blue exclusion. Data correspond to the mean ± standard deviation of two independent experiments.

With the aim of possible therapeutic applications using resveratrol derivatives in mind it was important to evaluate the specificity of cytoxicity towards normal cells. Hence, we evaluated the effect of potent derivatives on the proliferation of intestine epithelium IEC18 cells. The results shown in Figure 7 indicate no significant toxic effect of compounds **2**–**4** at 30 µM, except for compound **5** (presence of vinyl group in position 4). At higher concentration (100 µM) all compounds, including resveratrol, slightly inhibit cell proliferation, but much less than with the tumor SW480 cell line.

#### 2.2.4. Comparison of Resveratrol Analogs on Cytotoxicity of Colorectal Tumor Cells and on Hepatoblastoma Cells

To have an overall view of the mechanisms involved in the inhibitory effect of the compounds, we performed a concentration-dependent analysis of the cytotoxicity evaluated by the crystal violet method. The crystal violet assay was chosen for the screening of the dose-effect of numerous molecules despite its lower sensitivity compared to some other cytotoxicity methods [73]. The results are presented as IC_50_ values. These IC_50_ values have been determined both on human tumor colorectal SW480 cell line and on human hepatoblastoma HepG2 cell line (Table 1). All tested molecules have lower IC_50 _than resveratrol towards SW480 cell line. Compounds **2** and **4** show a similar activity, indicating that the additional hydroxy group does not increase the activity of the stilbene. Comparison of the IC_50_ values between compounds **2** and **10** confirm the importance of the position 4 of the phenolic group [30,31]. In the series of ferrocenylstilbene analogs, compound **17** without a free phenolic function is the most active. This may be explained by a better lipophilicity due to the ester group while the antitumor activity can be attributed to the ferrocenyl moiety. Five of the most active derivatives (compounds **1**, **2**, **5**, **6** and **8**) have been subsequently tested on the HepG2 cell line (Table 1). Compounds **1**, **2**, **5** and **6** exhibit a lower potency on HepG2 than on SW480 cell line. Compounds **7** and **10** are the least active towards SW480 cells. Interestingly, compounds **5** (vinyl group in position 4') and **8** (carbinol group in position 3 and methoxy in position 4') exhibit a higher activity towards SW480 cell lines than HepG2 cell lines, while the bromine in position 4' (compound **6**) has an opposite effect. In the case of compound **8**, its metabolism by HepG2 cells may explain its weaker activity towards these cells. The difference between the resveratrol IC_50_ cytotoxicity value (68.1 µM), (Table 1) and its inhibitory efficiency (30 µM) on cell proliferation (Figure 5) towards SW480 cell line would be attributed to the difference in the experimental approaches. 

**Table 1:** Compared IC_50_ values of stilbene and ferrocenyl derivatives towards cell proliferation of SW480 and of HepG2 cell lines. For technical informations, see experimental procedure (Cell proliferation assays).

Compound Number	Compound Name	SW480 IC_50_ (μM)	HepG2 IC_50_ (μM)
	*E*-resveratrol	68.1 ± 5.5	57.3 ± 8.1
1	*E*-4-hydroxystilbene	18.6 ± 3.2	27.6 ± 5.0
2	*E*-4-hydroxy-4'-methoxystilbene	14.7 ± 2.1	26.3 ± 3.2
3	*E*-4-hydroxy-3',5'-dimethoxystilbene	16.1(± 2.9	Not Tested
4	*E*-4,4'-dihydroxy-4'-methoxystilbene	15.0 ± 0.9	Not Tested
5	*E*-4-hydroxy-4'-vinylstilbene	21.4 ± 0.3	33.2 ± 6.2
6	*E*-4-bromo-4'-hydroxystilbene	25.3 ± 2.4	18.6 ± 0.2
7	*E*-4-hydroxy-3,3',4',5'-tetramethoxystilbene	38.2 ± 0.7	Not Tested
8	*E-*3-carbinol*-*4-hydroxy-4'methoxystilbene	25.7 ± 2.1	77.7 ± 4.1
10	*E*-3-hydroxy-4'-methoxystilbene	81.7 ± 3.7	Not Tested
—	Ferrocene	>100	>100
15	*E*-(4-vinylphenol)-ferrocene	25.5 ± 1.6	40.2 ± 4.3
16	*(E,E)-*1,1'-bis(4-vinylphenol)-ferrocene	>100	>100
17	*(E,E)-*1,1'-bis[(1-*p*-toluoyloxy-4-vinyl)benzene]-ferrocene	5.9 ± 0.1	5.1 ± 0.2

#### 2.2.5. Effect of Resveratrol Isosteres Bearing a Ferrocenyl Moiety. Determination of IC_50_ Values

Ferrocenyl derivatives were tested on cancerous SW480 and HepG2 cell lines and the IC_50_ values are reported in Table 1. Compound **17** shows the highest inhibitory activity in both cell lines with a very low IC_50_ value (5.9 µM), more than 10-fold higher compared to the resveratrol activity. Ferrocene used as a control does not induce any cytotoxic effect against SW480 cell line. Compound **16** (a deprotected version of compound **17**) shows a higher IC_50_ value (IC_50_ > 100 µM) than compound **17**. This data can be explained by the low solubility of **16** in DMSO in the cell medium. *E*-(4-vinylphenol)ferrocene (**15**), the closest isostere of resveratrol presented in this study shows a similar antiproliferative activity to resveratrol despite a lower solubility in the medium.

## 3. Experimental

### 3.1. General Experimental Procedures

Wittig reactions were performed under an inert atmosphere of argon using conventional vacuum-line and glasswork techniques. THF was degassed and distilled by refluxing over sodium and benzophenone under argon. The organic reagents were received from commercial sources and used without further purification. Separations by flash chromatography were performed on silica gel (230–400 mesh). ^1^H-NMR, ^13^C-NMR and ^31^P-NMR spectra (δ, ppm) were recorded in CDCl_3 _solutions on a Bruker 300 MHZ spectrometer, HRMS on MicroTOF Q-Bruker (ESI ionization). Spectroscopic analyses were performed at the Pôle de Chimie Moléculaire de l’Université de Bourgogne

### 3.2. Precursors of Ferrocenyl-Stilbene Analogs

*4-Toluoyloxybenzylic alcohol* (**23**): To a mixture of 4-hydroxybenzylic alcohol (**21**, 100 g, 80.65 mmol) and potassium carbonate (13.4 g, 96.6 mmol) in acetone (300 mL) was added over 30 min at 0 °C a solution of *para*-toluoyl chloride (16 mL, 121 mmol) in acetone (100 mL). Then, the mixture was refluxed for 6 h. After cooling, the inorganic salts were filtrated and washed with acetone. The solvent was removed under vacuum and the crude product was purified by chromatography (EtOAc/heptane: 1/4) to give pure 4-toluoyloxybenzylic alcohol (**23**) in 47% yield. ^1^H-NMR δ (ppm): 2.48 (s, 3H, CH_3_), 4.75 (d, 2H, CH_2_), 7.23 (d, 2H, Ar-H), 7.33 (d, 2H, Ar-H), 7.45 (d, 2H, Ar-H), 8.11 (d, 2H, Ar-H); ^13^C-NMR δ (ppm): 21.75 (CH_3_), 64.87 (CH_2_), 117.46–144.52 (Ar-C).

*4-Toluoyloxybenzylic bromide* (**24**): To a mixture of **23** (9 g, 37.70 mmol) and triphenylphosphine (14.9 g, 56.53 mmol) in CH_2_Cl_2_ (150 mL) was added a solution of N-bromosuccinimide (10 g, 56.53 mmol) in CH_2_Cl_2_ (100 mL). After stirring for one hour, the mixture was poured into a separatory funnel and was washed with water. The organic phase was dried over MgSO_4_. After removal of the solvent, the crude product was crystallized from ethanol (64%). ^1^H-NMR δ (ppm): 2.39 (s, 3H, CH_3_), 4.45 (d, 2H, CH_2_), 7.12 (d, 2H, Ar-H), 7.24 (d, 2H, Ar-H), 7.38 (d, 2H, Ar-H), 8.01 (d, 2H, Ar-H); ^13^C-NMR δ (ppm): 21.76 (CH_3_), 32.74 (CH_2_), 122.14, 126.61, 129.32, 129.78, 130.24, 135.29, 144.57, 150.96 (Ar-C), 165.04 (C=O).

*4-Toluoyloxybenzyltriphenylphosphonium bromide* (**20**): A mixture of **24** (18.7 g, 33 mmol) and triphenylphosphine (9.7 g, 36.3 mmol) in toluene (50 mL) was refluxed for five hours. The reaction mixture was cooled down to room temperature and a first crop of product was collected by filtration. The filtrate was then refluxed for five additional hours and a second crop of product precipitated. Two other crops were then collected and the combined fractions were crystallized from ethanol (86%). ^1^H-NMR δ (ppm): 2.37 (s, 3H, CH_3_), 5.47 (d, 2H, CH_2_), 6.90 (d, 2H, Ar-H), 7.12 (d, 2H, Ar-H), 7.22 (d, 2H, Ar-H), 7.66 (m, 15H, Ar-H phosphonium), 7.96 (d, 2H, Ar-H); ^13^C-NMR δ (ppm): 21.13 (CH_3_), 60.48 (CH_2_), 126.55 (Ar-C), 129.45, 130.28 (Ar-C phosphonium), 132.81, 134.54, 134.68, 135.05, 135.09, 144.76, 151.20 (Ar-C), 165.04 (C=O); ^31^P-NMR δ (ppm): 23.50 (s, 1P).

### 3.3. Ferrocenyl-Stilbene Analogs **15**–**17** and **25**

*E-[(1-paratoluoyloxy-4-vinyl)benzene]-ferrocene* (**25**): Under argon atmosphere, butyllithium (1.6 M, 2.8 mL, 4.48 mmol) was slowly added to a solution of 4-toluoyloxybenzyltriphenylphosphonium bromide (**20**, 2.5 g, 4.41 mmol) in THF (40 mL) at −78 °C. The resulting solution was allowed to warm at room temperature. A solution of ferrocenecarbaldehyde [59] (**18**, 0.95 g, 4.41 mmol) in THF (15 mL) was added dropwise and the reaction mixture was then stirred overnight. Ice-cold water (500 mL) was added and the mixture stirred for an additional hour. The aqueous layer was extracted with ethyl acetate; the combined organic layers were washed with water and dried over MgSO_4_. After evaporating the solvent, 52% of a crude mixture of isomers *Z* and *E* was isolated. The *E* isomer was isolated by chromatography (heptane/EtOAc: 9/1), yield 34%. ^1^H-NMR δ (ppm): 3.33 (s, 3H, CH_3_), 4.00 (s, 5H, Fc-H), 4.14 (t, 2H, Fc-H), 4.38 (d, 2H, Fc-H), 6.67 (d, 1H, *^3^J* = 16.65 Hz, =CH), 6.85 (d, 1H, *^3^J* = 16.65 Hz, =CH), 7.05 (d, 2H, Ar-H), 7.23 (d, 2H, Ar-H), 7.38 (d, 2H, Ar-H), 7.94 (d, 2H, Ar-H); ^13^C-NMR δ (ppm): 21.4 (CH_3_), 60.0, 65.9, 66.8 (Fc-C), 119.8, 124.2, 124.4, 125.1, 127.1, 127.9, 131.2, 135.09, 143.3, 148.3 (Ar-C), 165.3 (C=O); C_26_H_22_FeO_2_ (MW 422.01). HRMS (ESI): *m/z* 422.09629 [M]^+^, calculated mass 422.09637 (σ = 0.2 ppm).

*(E,E)-1,1'-bis[(1-paratoluoyloxy-4-vinyl)benzene]-ferrocene* (**17**): Under an argon atmosphere, butyl lithium (1.6 M, 5.6 mL, 8.96 mmol) was slowly added to a solution of 4-toluoyloxy-benzyltriphenylphosphonium bromide (**20**, 5 g, 8.82 mmol) in THF (80 mL) at −78 °C. The resulting solution was allowed to warm at room temperature. A solution of ferrocene-1,1'-dicarbaldehyde [59] (**19**, 0.95 g, 4.41 mmol) in THF (15 mL) was added dropwise and the reaction mixture was stirred overnight. Ice-cold water (500 mL) was added and the mixture was stirred for an additional hour. The aqueous layer was extracted with ethyl acetate; the combined organic layers were washed with water and dried over MgSO_4_. After evaporating the solvent, 47% of a crude mixture of *EE/E*Z/Z*Z* isomers was obtained. The *EE* isomer was isolated by chromatography (heptane/EtOAc: 9/1), yield 25%. ^1^H-NMR δ (ppm): 3.41 (s, 6H, CH_3_), 4.28 (t, 4H, Fc-H), 4.48 (d, 4H, Fc-H), 6.63 (d, 2H, *^3^J* = 15.09 Hz, =CH), 6.81 (d, 2H, *^3^J* = 15.09 Hz, =CH), 7.11 (d, 4H, Ar-H), 7.28 (d, 4H, Ar-H), 7.41 (d, 4H, Ar-H), 8.09 (d, 4H, Ar-H); ^13^C-NMR δ (ppm): 22.3 (CH_3_), 67.9, 68.1, 70.4 (Fc-C), 121.7, 124.1, 124.5, 125.1, 127.2, 127.7, 131.3, 143.5, 148.0 (Ar-C), 164.3 (C=O); C_42_H_34_FeO_4_ (MW 657.18). HRMS (ESI): m/z 658.17693 [M]^+^, calculated mass 658.18018 (σ = 4.8 ppm).

*E-(4-Vinylphenol)-ferrocene* (**15**): To a solution of **25** (0.51 g, 1.1 mmol) in MeOH (15 mL) were added pellets of KOH (0.17 g, 3.2 mmol). The mixture was stirred for one hour at 30 °C. The reaction was quenched by addition of water (15 mL) and the solution was stirred for four hours. The solution was acidified to pH = 2 by concentrated HCl and then treated with aqueous NaHCO_3_ solution (5%) to reach pH = 4. The ferrocene derivative **15** was extracted with ether. The combined organic layers were dried over MgSO_4_ and after removal of the solvent, the compound **15** was isolated, yield 92%. ^1^H-NMR δ (ppm): 4.25 (d, 4H, Fc-H), 4.27 (t, 2H, Fc-H), 4.43 (t, 2H, Fc-H), 4.60 (t, 1H, Fc-H), 4.74 (t, 1H, Fc-H), 4.79 (t, 1H, Fc-H), 6.36 (d, 1H, *^3^J* = 16.08 Hz, =CH), 6.71 (d, 1H, *^3^J* = 16.08 Hz, =CH), 6.79 (d, 2H, Ar-H), 7.28 (d, 2H, Ar-H); ^13^C-NMR δ (ppm): 68.7, 69.1, 69.6, 73.3 (Fc-C), 115.8, 125.3, 127.6, 130.6, 157.8, (Ar-C); C_18_H_16_FeO (MW 304.05). HRMS (ESI): *m/z* 304.05368 [M]^+^, calculated mass 304.05452 (σ = 2.7 ppm).

*(E,E)-1,1'-bis(4-Vinylphenol)ferrocene* (**16**): Following the procedure described above, compound **16** was obtained from **17**; 88%. ^1^H-NMR δ (ppm): 4.74 (d, 4H, Fc-H), 4.25 (t, 4H, Fc-H), 6.49 (s, 4H, =CH), 6.56 (d, 4H, Ar-H), 7.06 (d, 4H, Ar-H), 8.13 (s, 2H, OH); ^13^C-NMR δ (ppm): 67.1, 69.3, 82.3, (Fc-C), 114.4, 126.7, 127.3, 131.5, 159.1, (Ar-C); C_26_H_22_FeO_2_ (MW 422.09). HRMS (ESI): *m/z* 422.09588 [M]^+^, calculated mass 422.09765 (σ = 4.2 ppm).

### 3.4. Biological Methods

#### 3.4.1. Cell Culture

The human colon carcinoma cell line SW480 obtained from ATCC (American Type Culture Collection, Manassas,VA, USA) was cultured in RPMI-Medium with 10% fetal bovine serum (FBS) and 1% antibiotics. Human derived hepatoblastoma cell line HepG2 was obtained from the ECACC (European collection of cell culture, Salisbury, UK) and non-cancerous IEC18 cells from ileum epithelium of *Rattus norvegicus* (ATCC) were grown in monolayer culture system and maintained in phenol-red Dulbecco’s Modified Eagle’s Medium (DMEM) supplemented with 2 mM l-glutamine, 1% non-essential amino-acids, and 10% FBS (v/v) in a humidified atmosphere of 5% CO_2_ at 37 °C.

#### 3.4.2. Cell Viability Assays

Proliferation inhibition assays were performed in 24-well plates in triplicate, and each experiment was conducted two to three times. 30,000 cells were seeded per well, after 24 h cells were incubated in medium containing either 0.1% dimethylsulfoxide-solubilized *trans*-resveratrol, resveratrol derivatives, or 0.1% dimethylsulfoxide (DMSO) only as control. After 48 h, cells were harvested and the number of live cells was quantified using the trypan blue exclusion test which is based on the ability of a viable cell with an intact membrane to exclude trypan blue dye using a haemocytometer in microscopic counting. Results were expressed as percentage of control values. 

#### 3.4.3. Cell Proliferation Assays

After 48 h of incubation at 37 °C, medium was carefully removed from wells and the plates were washed gently with PBS 1X warmed at room temperature. Then the crystal violet solution was added and incubated for 10 min. Thereafter, plates were washed several times with tap water. The nucleus-incorporated crystal violet was dissolved using a sodium citrate solution and plates were agitated on orbital shaker until the color became uniform with no areas of dense coloration at the bottom of wells. The absorbance was read on each plate at 540 nm with a spectrophotometer (Dynex MRX-TC Revelation, Manassas, VA, USA). The absorbance is proportional to the relative density of cells adhering to multi-well dishes in regard to the absorbance of control well-plate (5% DMSO). After 48 h, IC_50_ values were determined by performing 0.75 to 100 µM treatments and the IC_50_ values were obtained after parametric regressions on the percentages of viable cells *versus* the control.

#### 3.4.4. Cell Cycle Analysis

Cell cycle analysis was performed as described previously [67,74,75]. Briefly, cells were seeded 24 h before treatment into 25 cm^2^ flasks. After treatment, the detached and adherent cells were pooled, fixed with ethanol, and stained with propidium iodide (PI) for subsequent analyses with a CyFlow Green flow cytometer and the fluorescence of PI was detected above 630 nm. For each sample 20,000 cells were acquired. Furthermore, data were analyzed with the MultiCycle software (Phoenix Flow Systems, San Diego, CA, USA); the x-axis corresponds to the DNA content and the y-axis to the number of cycling cells. The maximum value on the y-axis is inversely proportional to the altered cells level (non-cycling cells) which is excluded by gating.

## 4. Conclusions

While *trans*-resveratrol is considered a promising molecule for fighting cancer [76], a wide range of synthetic resveratrol analogs are potentially more active than *trans*-resveratrol. Some of these new synthetic molecules have interesting effects. Compounds **2** and **17** are the most active, while compounds **10** and **16** show the lowest activity. The comparison between compounds **16** and **17** indicates that the presence of a protecting group lead to a better efficacy which could be due to a better solubilisation in DMSO. It appears that the lack of substituents at position 3 and 5 (compound **1**) leads to a better inhibitory effect. Moreover, a limited number of methoxy groups (compounds **2**, **3** and **4**) provides better lipophilic properties. In most cases, the efficacy of the synthetic compounds is lower towards liver derived HepG2 cells than towards colorectal SW480 cells, except for compound **6** and mostly **17**, which is the most powerful derivative. These differences can be explained by the high xenobiotic metabolizing activities of HepG2 cells. Furthermore, the lack of effect on non-tumor cells (IEC18 intestinal epithelium cells) demonstrates the selectivity of these molecules for cancer cells, which is an important aspect for potential therapeutic applications. Concerning the possible targets of resveratrol analogs, an inhibition of the TNF alpha-induced activation NFkB by polyhydroxylated resveratrol derivatives *i.e*., the hexahydroxystilbene in leukemia HL60 cells has been reported [70]. In terms of the structure-activity relationship, it appears that in order to obtain an inhibitory effect, the chemical parameters are the following: (a) the presence of a hydroxy group in position 4; (b) an increased inhibitory effect by the presence of a methoxy group (a decrease of the polar character leading to an increase in lipophilicity); (c) the lack (or masked form) of other hydroxy groups. In addition, (*E,E)*-1,1'-bis[(1-*para*-toluoyloxy-4-vinyl)benzene]ferrocene (**17**) a new compound, shows the highest efficacy. 

## Supplementary Materials

Supplementary materials can be accessed at: http://www.mdpi.com/1420-3049/19/6/7850/s1.
