# Supplementary material for: Inhibition of Cancer Derived Cell Lines Proliferation by Synthesized Hydroxylated Stilbenes and New Ferrocenyl-Stilbene Analogs. Comparison with Resveratrol
Source: Molecules. 2014 Jun 11;19(6):7850–68. doi: 10.3390/molecules19067850 (PMC6271691; doi:10.3390/molecules19067850)
Supplement: Supplementary file 1 [file molecules-19-07850-s001.pdf]

Supplementary Information

Figure S1. SM. Flow cytometry measurements of the effect of stilbene derivatives on the cell cycle phases of SW480 cell line.

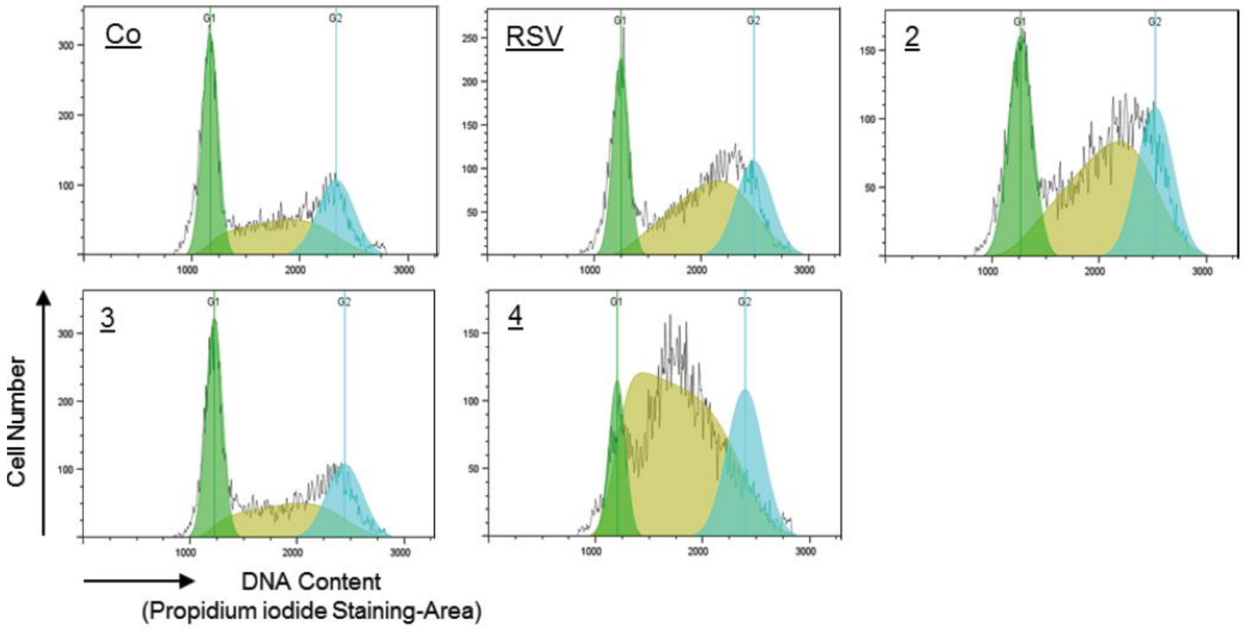

Cells were grown for 48 h in the presence or not (control) of resveratrol (RSV) at 30 µM or of stilbene derivatives (30 µM). After treatment, nuclear DNA was labeled with propidium iodide, then cell cycle phases were analysed by flow cytometry.
